# Competency-Based Assessment for Clinical Supervisors: Design-Based Research on a Web-Delivered Program

**DOI:** 10.2196/resprot.3893

**Published:** 2015-02-27

**Authors:** Rachel Bacon, Lauren Therese Williams, Laurie Grealish, Maggie Jamieson

**Affiliations:** ^1^School of Public Health and NutritionBruce ACTAustralia; ^2^Griffith Health InstituteGriffith UniversityGold CoastAustralia; ^3^School of Public Health and NutritionFaculty of HealthUniversity of CanberraBruce ACTAustralia

**Keywords:** competency-based education, preceptorship, e-learning, pedagogy, constructivist, dietitian

## Abstract

**Background:**

Clinicians need to be supported by universities to use credible and defensible assessment practices during student placements. Web-based delivery of clinical education in student assessment offers professional development regardless of the geographical location of placement sites.

**Objective:**

This paper explores the potential for a video-based constructivist Web-based program to support site supervisors in their assessments of student dietitians during clinical placements.

**Methods:**

This project was undertaken as design-based research in two stages. Stage 1 describes the research consultation, development of the prototype, and formative feedback. In Stage 2, the program was pilot-tested and evaluated by a purposeful sample of nine clinical supervisors. Data generated as a result of user participation during the pilot test is reported. Users’ experiences with the program were also explored via interviews (six in a focus group and three individually). The interviews were transcribed verbatim and thematic analysis conducted from a pedagogical perspective using van Manen’s highlighting approach.

**Results:**

This research succeeded in developing a Web-based program, “Feed our Future”, that increased supervisors’ confidence with their competency-based assessments of students on clinical placements. Three pedagogical themes emerged: constructivist design supports transformative Web-based learning; videos make abstract concepts tangible; and accessibility, usability, and pedagogy are interdependent.

**Conclusions:**

Web-based programs, such as Feed our Future, offer a viable means for universities to support clinical supervisors in their assessment practices during clinical placements. A design-based research approach offers a practical process for such Web-based tool development, highlighting pedagogical barriers for planning purposes.

## Introduction

### Support for Supervisors to Assess Clinical Competence

Within the dietetics profession, students are required to complete 20 weeks of placement, with half of that time spent in developing and demonstrating competence in individual case management [1]. The assessment of the clinical competence of student dietitians is a shared responsibility between the university and the health sector [1], with the assessments made by site supervisors during clinical placements providing a key source of evidence of student competence [2]. The difficulties faced by site supervisors in assessing student performances during clinical placements are clearly reported in the literature [3,4]. Clinicians therefore need to be supported by universities to use credible and defensible assessment practices [5]; however, the geographical distribution of placement sites prohibits face-to-face education of all supervisors.

### Web-Based Delivery

Web-based delivery of education to support clinical supervisors has been successfully used by the professions of medicine, nursing, and physiotherapy [6-9]. The Web-based mode transcends geographical and time constraints [10] and may be more accessible to clinicians, particularly those in rural or community-based settings who may be sole practitioners within a multidisciplinary team [11]. Web-based delivery provides an efficient means to share resources and avoid duplication [12]. Professional development delivered via the Web has been shown to achieve equivalent outcomes (satisfaction, knowledge retention, and change in practice) when compared to face-to-face delivery [13,14].

### Pedagogy

When developing a Web-based learning program, both the discipline-specific content and the learning process need to be considered. Constructivist pedagogy, in which learners construct their own meaning by forming connections through collaboration and reflection between their prior knowledge and new experiences (authentic real-world problems), has been recommended for Web-based delivery [15,16]. Collaboration can be supported within a virtual community using a central online discussion forum [17]. This learner-centered approach to Web-based education allows participants to be independent self-paced learners and to select learning content in a way that meets their learning style [16,17]. Rowe and Rafferty [18] have demonstrated improved user engagement with Web-based learning by self-regulated learning strategies such as activation of prior knowledge, self-monitoring, and reflections. There is evidence to suggest video-based learning material may improve learner engagement [9,19-21]. Effective Web-based delivery must also consider the usability and accessibility of the program [22,23].

### Objective

Programs to educate supervisors in the use of more credible and defensible assessment practices are currently non-existent. This paper explores the potential for a Web- and video-based constructivist tool to support clinical supervisors to use credible and defensible assessment practices during clinical placements. The program aims to use authentic video-based learning material and metacognitive activities such as self-monitoring and reflection to support clinical supervisors to transform their assessment practices. The study also considers the interdependence between pedagogy, usability, and accessibility.

## Methods

### Design-Based Research

The Web-based program “Feed our Future” was developed using a design-based research approach adapted from Wang and Hannifin [24]. This approach has been used in the design of technology-enhanced learning environments for the way in which it advances design, research, and practice concurrently [25]. Design-based research addresses a practical problem in context, is informed by theory, and is refined through an iterative process of formative feedback and reflection in consultation with participants [25]. In the final stage of product development in design-based research, the intervention is pilot-tested and evaluated. This stage is then used to inform final revisions of the program [24].

### Stage 1: Program Development

In October 2012, research consultation and initial development of the program commenced concurrently.

#### Research Consultation

Research presented in the publication, “Credible and defensible assessment of entry-level clinical competence: Insights from a modified Delphi study” [26], informed the development of the professional content of the program. This research was conducted with a panel of experienced clinical supervisors (potential end-users) and explored the issues of judgment and subjectivity in the assessment of health professional competence. The paper includes a focused literature review on credible and defensible competency-based assessment practices including the need for a shared definition of competence [27], clearly defined standards [28], a global approach to assessment [29], consideration of the learning context [30,31], multiple sources of evidence [32], and the need for an interpretive community of assessors [33].

#### Development of the Prototype

An interview with Professor Sue Ash, a member of the original taskforce that developed the dietetic competency standards in 1994 [34], and participated in their reviews [35,36], was recorded as expert opinion. This recording was incorporated into the program to provide clarity on the definition and application of the competency standards within the dietetics profession.

Evidence suggests that resources to support assessments such as visual representation of entry-level performance may increase the consistency of supervisor assessments [37]. As an outcome from the research consultation, 11 video recordings of authentic dietetic student-client consultations were produced for the program (mean duration 60 minutes; residential aged care and outpatient settings), with corresponding assessments of each student’s performance made by the panel of experienced clinical supervisors [26].

Information technology (IT) expertise from an academic was sought to select an appropriate delivery platform. Consideration was given to budget and timeline, security, usability, incorporation of different file types, with particular considerations of video recordings, and capacity to provide feedback to participants on their learning.

The first prototype of Feed our Future was completed within 8 months using the website builder WIX as the delivery platform. The planned learning outcomes for the program were for supervisors (1) to feel more confident in their approach to assessment, and (2) to use credible and defensible competency-based assessment practices. The program comprised four learning modules, each including questions to consider, problem-based learning and self-monitoring activities, key concepts, and suggested readings. A pre-program quiz, a post-program quiz, a discussion forum, and a practice capstone module were included.

#### Formative Feedback

Feedback obtained during the Feed our Future program development included several sources. An advisory group comprised of industry, academic, student, consumer, and regulatory representation provided direction on the research and the development of the Web-based program. Potential end users trialed the prototype and provided feedback via a market stall/booth established at the Annual National Conference of the Dietitians Association of Australia (DAA) in May 2013. The DAA’s Board of Directors also reviewed the program.

### Stage 2: Pilot Test and Evaluation

#### Participants

A purposeful sample of nine dietitians located in a variety of health care sites and involved in the University of Canberra’s clinical placement program was invited, via email, to participate in this study. These potential end-users were provided with access to the password-protected website and asked to pilot-test the program over a 4-week period. The Human Research Ethics Committee (HREC) of the University of Canberra approved the study protocol (12-209) that conformed to the provisions of the Declaration of Helsinki.

#### Data Generated From Feed Our Future

Data generated as a result of user participation during the pilot test including participation rates and outcomes from the pre-test, discussion forum, multiple choice quiz, and the post-test, were reviewed. In the pre-test and post-test, participants were asked to (1) rate their level of confidence with assessing a student’s competence during his/her clinical placement using a 10-point scale (1=not at all confident; 10=extremely confident), (2) rate a student’s performance as observed from a video recording (the method of assessment is described elsewhere [26]), and (3) provide a qualitative description of how they would ensure their assessment of a student’s competence during his/her clinical placement was credible and defensible. Content analysis was used to analyze the qualitative responses from the discussion forum, informed by the focused literature on credible and defensible assessment practices described in the research consultation section [26].

#### Qualitative Evaluation

User experiences during the pilot test were explored using an interpretivist qualitative approach. During the pilot test, users were invited to reflect on a series of questions to be discussed at a later interview. Interviews were held in a focus group for those who could attend (n=6) and in the format of individual interviews, via telephone, for the remainder (n=3). Focus groups were chosen to make use of group dynamics to stimulate discussion in a secure environment [38]. The focus group and individual interviews were facilitated by the primary researcher and began with a scripted introduction outlining the research and ethical considerations. Users provided informed signed written consent that included permission for their interview to be audio-recorded. In the focus group session, a research assistant was employed as a scribe.

The interview questions were developed by the first author in consultation with LW and MJ and covered (1) the overall experience of using the Feed Our Future program, (2) what they learned, (3) whether and in what way their thinking had been challenged, (4) whether it had prompted them to change the way they assessed students on their clinical placement, and (5) suggestions to improve the program. Users were also asked to describe their workplace and experience with supervising and assessing students up to the time of viewing the program.

Recordings were audiotaped, transcribed verbatim by two researchers, and crosschecked for accuracy to maintain the integrity of user responses. Transcripts were analyzed independently by the primary researcher and one research assistant with themes highlighted using van Manen’s highlighting approach to thematic analysis [39]. Pedagogical themes arising from the focus group and individual interviews were compared and found to be similar enough to pool. Exemplar quotations illustrating each theme were identified.

## Results

### Stage 1: Program Development

#### Formative Feedback

Table 1 presents the formative feedback that was generated from the advisory committee, dietitians at the Dietitians Association of Australia’s (DAA) National Conference, and from the DAA Board of Directors.

**Table 1:** Formative feedback and subsequent refinement to the program.

Source	Feedback	Refinement
**Advisory Committee**		
	Learner-centered approach	No change recommended
	Professional content	Emphasized that competence cannot be assessed from a single performance.
	Use of authentic videos	Keep videos footage in context
	An interpretative community of assessors	Include a feedback session with insights from students^a^
**End users at DAA conference stall**		
	Need for aesthetic improvements^a^	Reduced the amount of text per page
		Removed images that did not add meaning
		Increase consistency across modules
		Added audio file introduction
		Provided a program overview
	Further supports required to improve navigation^a^	Included direction arrows on each page
		Added a file path to each page
		Made all modules accessible from the homepage
	Ongoing IT issues with playing videos^a^	YouTube videos also made accessible through Dropbox as .mp4 file type
		Added contact details for IT support on homepage
	Discussion forum not accessible on WIX^a^	External discussion forum added
**Dietitians Association of Australia (DAA) Board**		
	Program endorsement under consideration	Review by the Australian Dietetics Council pending^a^
	Approved for dissemination through the Dietetics Information and Nutrition Education Resource Database (DINER) accessible to all DAA members	
	Agreed to promote program through professional online newsletter to DAA members	

^a^These items need addressing; all other items were supported/addressed.

### Stage 2: Pilot Test and Evaluation

#### Participants

Of the nine users that pilot-tested Feed our Future, two were from rural and seven from urban locations, four worked in hospitals and five in community settings, five were experienced supervisors, two reported some experience, and two had little or no experience with supervising students.

#### Data Generated From Feed Our Future

Data generated by participants after pilot-testing the program Feed our Future are presented in Tables 2- 4. In the pre-test, the mean confidence level for users with their assessment approach (using a 10-point scale: 1=not at all confident; 10=extremely confident) was 5.75 (range 2-9). In the pre-test, only five out of eight users rated the student performance, as observed from the video recording, in a similar way to the panel of experienced supervisors (see Table 3). In their qualitative responses, only some concepts supporting credible and defensible competency-based assessment practices were identified by the users (see Table 4).

Although technical issues prevented some users from participating, the discussion forum was used for introductions and to share learning and reflections. The average score achieved from the multiple choice quiz by users was 86%. Technical issues delayed participants’ completion of the program and hence no results are available from the post-test.

**Table 2:** Data generated from Feed our Future: participation.

Program feature	Number of users (n=9) n (%)
Pre-test	8 (89%)
Forum	4 (44%)
Multiple choice quiz	7 (78%)
Post-test	0 (0%)

**Table 3:** Data generated from Feed our Future: pre-test results Question 2: assessment rating of student’s performance by users.

Rating scale used to assess student’s performance	Number of users who rated the performance at each stage (n=8) n (%)
Novice	1 (13%)
Intermediate/beginner^a^	5 (63%)
Entry-level competent	2 (25%)

^a^Consistent with the panel rating.

**Table 4:** Data generated from Feed our Future: pre-test results Question 3: content analysis from qualitative responses informed by focus literature review [26].

Competency-based assessment practice considered by users	Number of users (n=8) n (%)
Defined standards	7 (88%)
Global approach	2 (25%)
Supervisor collaboration	3 (37%)
Evidence-based	6 (75%)

#### Qualitative Evaluation

The analysis of interview transcripts revealed three pedagogical themes: (1) constructivist design supports transformative online learning, (2) videos make abstract concepts tangible, and (3) accessibility, usability, and pedagogy are interdependent.

##### Theme 1: Constructivist Design Supports Transformative Online Learning

Although the post-test was not completed by users due to technical issues, qualitative feedback from the focus group and personal interviews showed an increase in user confidence as demonstrated by this exemplar quote:

From doing this, I now feel like I would be able to confidently have a final clinical placement student.

Focus Group User # 3

The constructivist design assisted users to apply their learning as demonstrated by the exemplar quotes in Table 5. The program enabled users to compare their assessments of an individual student performance with those made by a panel of experienced supervisors. As one user commented:

We can use this process for moderation, if we have a number of different supervisors that watch a particular video, we could use it to make sure that our assessments are similar…

Personal Interview User #9

Through participation in the program, users achieved consensus in their understanding of entry-level performance.

**Table 5:** Constructivist design supports transformative online learning.

Pedagogical feature	Exemplar quote
Learner-centered approach	I found the program very accessible, I found it well structured, I found it sort of oriented towards self-learning, and that you could complete it in different parts. (Focus Group User #5)
Authentic problem-based learning activities	I sort of never really thought about how to apply the competencies, and the types of patients, the different wards that we have in the hospitals… doing that activity where it had each of the competencies broken down and how you’d apply them …I was like, ‘oh’ I can totally figure out how to do it…(Focus Group User #2)
Metacognitive activities	I suppose it just made me sort of reflect on my transition from being you know a student to a new grad and it made a bit more sense, being able to apply it [the competency standards] in different situations…(Focus Group User #3).

##### Theme 2: Videos Make Abstract Concepts Tangible

Users supported the use of video-based learning material:

you can read about it, but actually seeing the videos of an assessment, and knowing where they sit on the scale [from novice to expert]…you know we always want to see stuff in action.

Focus Group User #3

They commented that prior to completing the program they had found the learning content “difficult to apply” and “frustrating at times”. The users found that the video representations of the authentic student-patient consultations allowed their understanding of “entry-level” competence to become more tangible.

I really liked the videos that when, at the end of them would show the scale of where the students were, like from the beginning to the end.

Focus Group Participant #2

**Figure 1:**
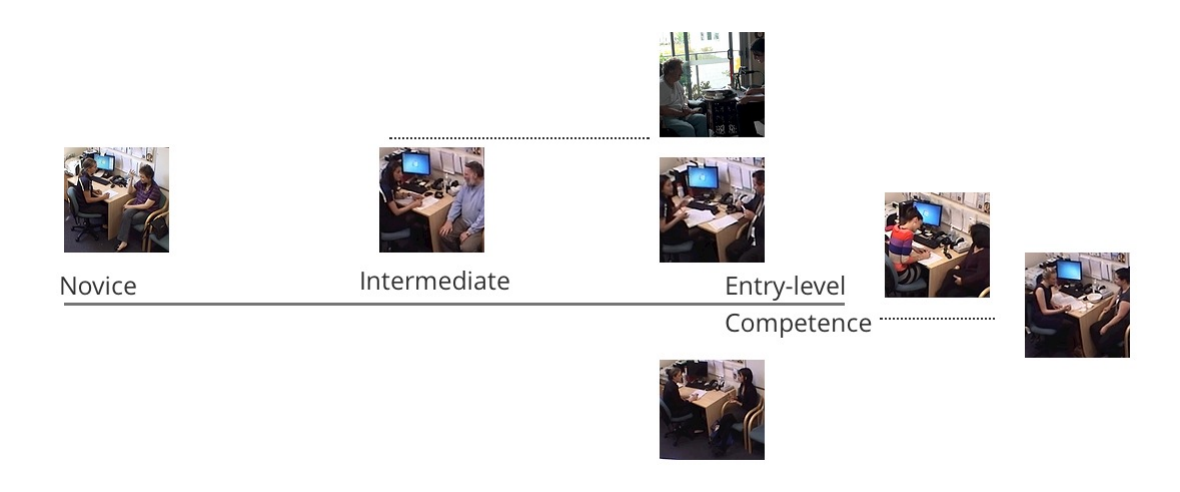
Visual representation of competency development using videos.

As demonstrated by Figure 1, organizing the videos on a scale helped the supervisors to distinguish between a novice, intermediate, and entry-level student performances.

##### Theme 3: Accessibility, Usability, and Pedagogy Are Interdependent

IT access and capacity at some worksites limited engagement with the program:

I’m computer literate but not really up-to-speed with some technological advances I suppose. I was a bit frustrated with some of those things…I suppose once I get annoyed with something I’m not inclined to go back.

Personal Interview User #7

Table 6 summarizes accessibility and usability barriers experienced by users and presents revisions made to improve the program.

### Product Release and Dissemination

**Figure 2:**
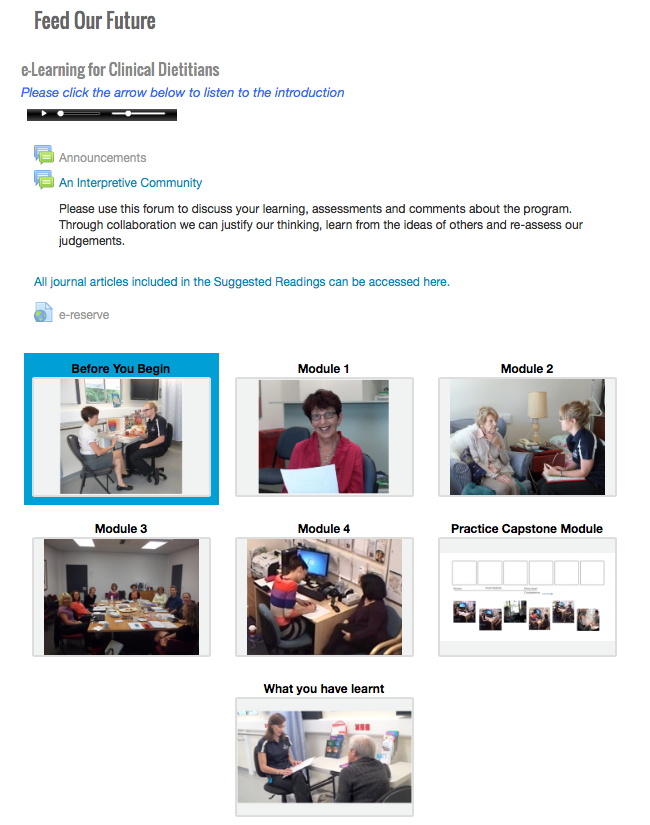
Final interface home page.

**Figure 3:**
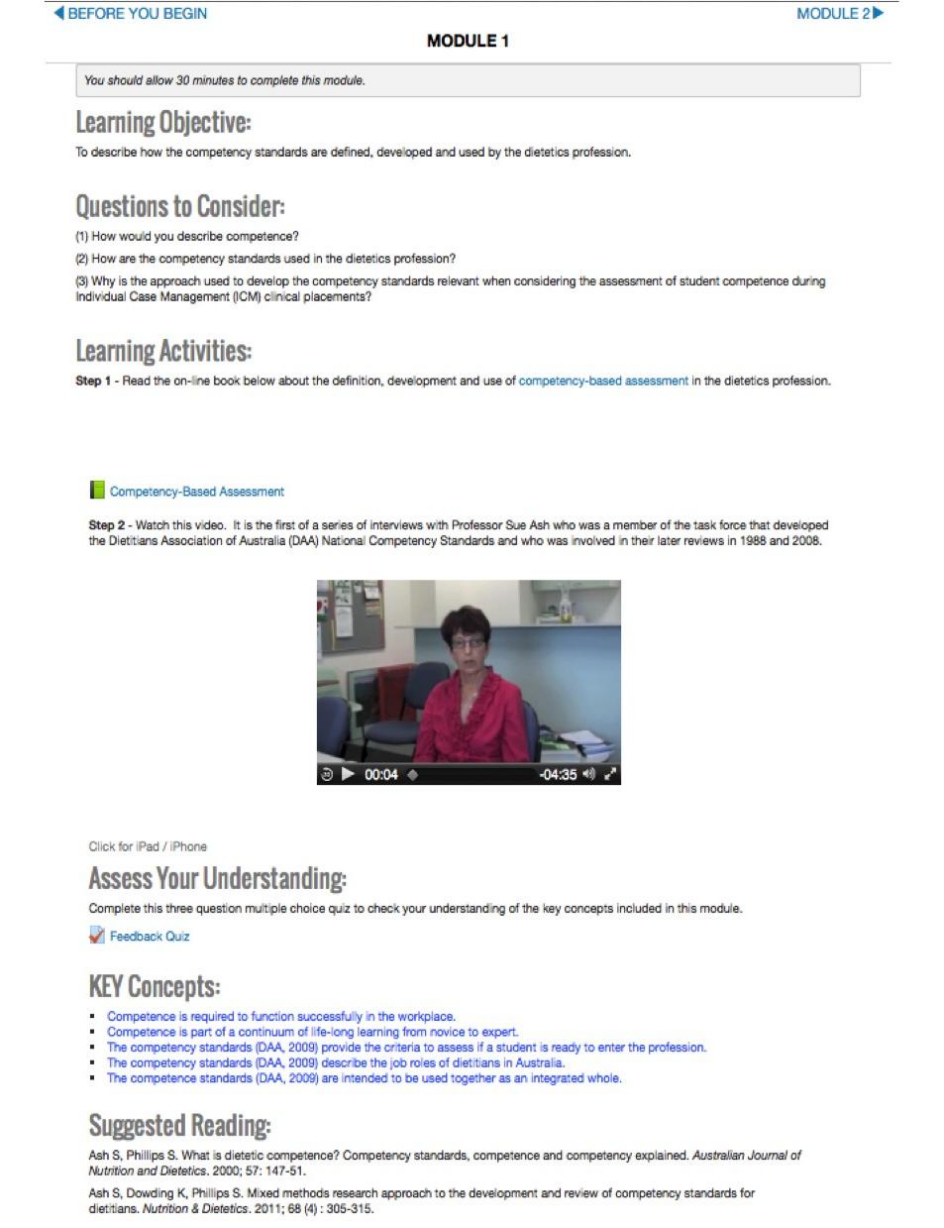
Final interface learning modules.

**Figure 4:**
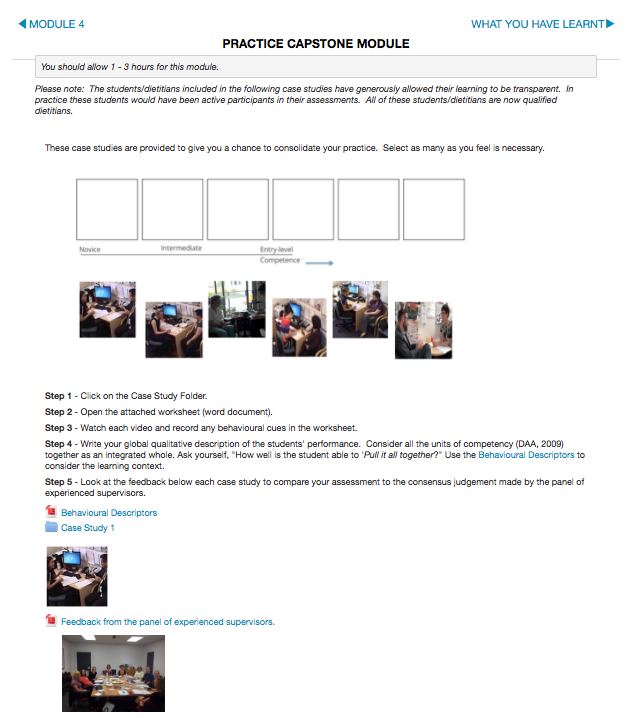
Final interface practice modules.

Table 7 presents the final learning content for Feed our Future. Figures 2-4 present screenshots of the final interface.

**Table 6:** Program features: barriers and solutions.

Program feature	Barriers	Solutions
**Usability**		
	Shared computer workstations	Changed to university-hosted delivery platform that supported individual log-ins and was compatible with Internet Explorer.
	Internet browsers available at some worksites not compatible with delivery platform	
	Clearer expectations required for learning modules including time commitments	An introductory video and program outline (including endorsement, program description, learning outcomes, learning content, background, acknowledgements, evaluation processes, and certificate of completion) were added to the program.
**Authentic video-based learning material**		
	Security restrictions for YouTube videos	Change to university-hosted delivery platform with embedded videos. Alternative access made available through Dropbox. Videos saved in generic .mov version.
	Length of patient /student encounters reduced engagement	Videos edited and shortened; average 8.5 min. (range 0.58-18.17)
	Network capacity issues	
**Virtual online community**		
	Security restrictions prevented participation at some sites	Changed to university-hosted delivery platform with embedded discussion forum

**Table 7:** Learning content included in Feed our Future*.*

Time (minutes)	Learning objective	Learning experiences	Self-monitoring
15	Before you begin	About this program	
		Engage your prior knowledge / Pre-test evaluation	
		Introducing the learning modules	
30	To understand how the competency standards are defined, developed, and used by the dietetics profession	Reading: Competency-based assessment	Multiple choice quiz
		Video: Development of competency standards with Sue Ash	
30	To explain the relationship between context and competence	Reading: Competence and context	Reflection
		Video: A case example of a non-traditional setting	
		Video: Challenges of the future workforce with Sue Ash	
60	To apply unit 4 of the competency standards (DAA, 2009) in your clinical setting	Reading: Applying the competency standards-1	Reflection
		Problem-based learning activity: Entry-level competence in your clinical setting	
		Reading: Applying the competency standards–2	
90	To evaluate student performances from authentic student-client consultations using a credible and defendable approach to competency-based assessment	Reading: Applying the competency standards-3	Compare with assessments by experienced supervisors
		Scaffolded case study: Assess a video of an authentic client consultation	
60-180	To consolidate credible and defendable competency-based practices using authentic student-client consultations	Case studies: Assess videos of authentic client consultations	Compared with assessments by experienced supervisors
15	What you have learned	Post-test evaluation	
		Certificate of completion	
		References	

## Discussion

### Principal Results

This paper describes the development of the first research- and Web-based learning program to support clinical supervisors in their assessments of student dietitian competence during clinical placements. This case example demonstrates the value of a design-based research and consultative approach to developing a program. The use of a Web-based mode has the potential to disseminate expertise and research findings nationally, overcoming geographical and time boundaries, in the provision of continuing professional development to health practitioners who assess student performance.

### Comparisons With Prior Work

The results of the pilot test supported the pedagogical design of Feed our Future*.* The program encouraged independent self-paced learning and catered to different learning styles as recommended by Ng’ambi and Lombe [16]. Participants demonstrated new understandings that aligned with the programs’ learning objectives of the program through the use of authentic student-client consultations, problem-based learning activities, and reflections. Kyeong-Ju Seo and Engelhard [9] achieved similar results with their constructivist Web-based continuing education program for physiotherapy supervisors with their participants perceiving improvements in the quality of their clinical education skills and practices. The approach used in this study highlights the interdependence of pedagogical, usability, and accessibility considerations [22] with the iterative process and the end-user involvement facilitating the identification of barriers to effective educational outcomes.

Participants in the pilot test found the video recordings of student-client consultations to be helpful in learning about competency-assessment practices. Clinical vignettes in traditional face-to-face learning programs have been used to assist supervisors to gain a shared understanding of entry-level competence in physiotherapy [37]. When used in Web-based delivery, videos have been shown to help engage students and improve learning outcomes [20,21,40]. Maloney and colleagues [19] found learners preferred videos in comparison to other learning materials made available through a Web-based resource repository. Developing a Web-based program with a large number of videos (n=20) in Feed our Future was technically challenging. The decision to edit and divide the videos was driven by network capacity limitations, but Guo’s research [41] suggests that short (6-9 minute) videos also have pedagogical advantages.

Consistent with the findings of Cook and Steinert [14], users appreciated material that was relevant, well-organized, and had clear expectations including time commitments. The participation rates for the discussion forum in this study were low despite the fact that other studies have identified conversational discussion and social bonding as key factors for successful Web-based education [14]. This feature is also key to constructivist pedagogy [16] and aligns with the notion of an interpretive community of assessors [33]. Possible solutions to address the lack of engagement with discussions may include more active moderation on the forum, blended Web-based learning with face-to-face contact, a social media approach that conforms to workplace security restrictions, or more assistance with technical problems [14].

Technical barriers experienced in the pilot-testing of Feed our Future such as IT incompatibilities between organizations’ infrastructure, software and Internet browsers, security restrictions, and bandwidth limitations are not unique [42]. Universities have very few security restrictions and are able to use programs such as YouTube to achieve positive learning outcomes [43]. Awareness that this freedom may not be available in some health settings is required if effective Web-based programs are to be available for use by clinical supervisors working in these settings.

Feed our Future, like many programs [44,45], was developed on a limited budget. Lack of IT expertise, infrastructure, and associated software, were limitations to the development of this program. Two years was required to complete the design-based research approach. Despite the advantages, the development requirements for Web-based programs are more labor intensive than face-to-face delivery [44,45].

### Limitations

The research-based design and national consultation used for the development of this program was robust. The sample size and the qualitative design of the pilot test and evaluation, although consistent with similar studies [46,47], does not support generalization of the results. Rather, these findings have been used to inform and improve the innovative product. Due to the lack of comparison with other modes of delivery, conclusions cannot be drawn as to whether Web-based delivery was the preferred option by clinical supervisors. The design-based research approach, however, offers supporting evidence for Web-based pedagogical approaches [25]. Further research is required to measure whether the learning of participants translated into actual changes in their competency-based assessment practices, and to determine the uptake of the program nationally.

### Conclusion

Web-based programs, such as Feed our Future*,* offer a viable solution for universities to provide professional development to geographically dispersed clinical supervisors in preparation for their students’ clinical placements. A design-based research approach offers a practical process for Web-based tool development.

## Abbreviations

DAA: Dietitians Association of Australia
IT: information technology
